# Effect of HCV treatment response on insulin resistance: A systematic review and meta-analysis

**DOI:** 10.3892/etm.2019.8334

**Published:** 2019-12-16

**Authors:** Jing-Hong Hu, Ming-Ling Chang, Nai-Jen Liu, Chau-Ting Yeh, Tung-Jung Huang

Exp Ther Med 18: 3568-3578, 2019; DOI: 10.3892/etm.2019.7995

Subsequently to the publication of this article, the authors have realized that name of the fourth listed author was spelt incorrectly. This appeared as “Chu-Ting Yeh”, whereas the name should have been printed as “Ch**a**u-Ting Yeh”. The corrected name is featured above.

The authors regret this error in the affiliations, and apologize to the author concerned and to the readers for the inconvenience caused.

